# Investigation of Nonlinear Piezoelectric Energy Harvester for Low-Frequency and Wideband Applications

**DOI:** 10.3390/mi13091399

**Published:** 2022-08-26

**Authors:** Osor Pertin, Koushik Guha, Olga Jakšić, Zoran Jakšić, Jacopo Iannacci

**Affiliations:** 1 National MEMS Design Centre, Department of Electronics and Communication Engineering, National Institute of Technology, Silchar, Assam 788010, India; 2 Center of Microelectronic Technologies, Institute of Chemistry, Technology, and Metallurgy–National Institute of the Republic of Serbia, University of Belgrade, Njegoševa 12, 11000 Belgrade, Serbia; 3 Center for Sensors and Devices (SD), Fondazione Bruno Kessler (FBK), Via Sommarive, 18, I-38123 Trento, Italy

**Keywords:** nonlinear piezoelectric energy harvester, monostable, wideband, vibration energy harvester

## Abstract

This paper proposes a monostable nonlinear Piezoelectric Energy Harvester (PEH). The harvester is based on an unconventional exsect-tapered fixed-guided spring design, which introduces nonlinearity into the system due to the bending and stretching of the spring. The physical–mathematical model and finite element simulations were performed to analyze the effects of the stretching-induced nonlinearity on the performance of the energy harvester. The proposed exsect-tapered nonlinear PEH shows a bandwidth and power enhancement of 15.38 and 44.4%, respectively, compared to conventional rectangular nonlinear PEHs. It shows a bandwidth and power enhancement of 11.11 and 26.83%, respectively, compared to a simple, linearly tapered and nonlinear PEH. The exsect-tapered nonlinear PEH improves the power output and operational bandwidth for harvesting low-frequency ambient vibrations.

## 1. Introduction

Vibration Energy Harvesters (VEHs) harvest ambient mechanical energy to replace traditional batteries and enable self-powered devices. The mechanical vibration is converted into useful electrical energy using conversion mechanisms such as electrostatic, piezoelectric, and electromagnetic processes [1]. The availability of piezoelectric material, easy size reduction, accessible employment, low cost, and high power density make the Piezoelectric Energy Harvester (PEH) still the most popular VEH [2,3]. The piezoelectric effect in PEH converts the developed strain in piezoceramic films to electrical output. PEHs have been implemented in self-powered micro-devices such as wireless sensor nodes [4,5], wearable devices [6,7] and implantable devices [8,9], among many others. Conventional cantilever-based PEHs generate maximum output at the structure’s resonant frequency [10]. However, such resonance-based linear harvesters with a narrow frequency band are inadequate for real-time harvesting of broadband and varying ambient vibrations.

A relatively recent approach to vibration energy harvesting is the use of nanogenerators. Similar to mesoscopic/MEMS devices, they can be based on the piezoelectric effect [11,12]. The piezoelectric nanogenerator (PENG) relies on the piezoelectric potential generated by nanowires to generate electric energy. Another important family of nanoharvesters is the triboelectric nanogenerators (TENG) [13,14,15]. They are based on the triboelectric effect combined with electrostatic induction. Degradation of triboelectric material due to friction heat is a major disadvantage in triboelectric generators [16]. There is still a need for more research on alternative materials such as self-healable materials [17]. Chung et al. developed a hybrid piezoelectric and triboelectric generator with an origami structure to allow effective energy harvesting [18]. Nanogenerators proved themselves very useful in, e.g., biomedical applications, sensors, wearable and implantable devices, and generally self-powered microelectronic and MEMS devices. Nanogenerators have the virtue of smaller dimensions, wide bandwidths and versatility. Mesoscale piezoelectric harvesters may outperform the nanoscale ones in output generation due to the difficulties of nano devices to produce sufficient displacements at a small scale to effectively convert ambient frequencies as found in nature and generate considerable powers [19].

In recent years, researchers have proposed numerous approaches for broadening the operational bandwidth and improving the performance of the PEHs. Early research proposed energy-harvesting systems with several degrees of freedom (DoFs) based on multiple piezoelectric harvester modes that utilize multi-power peak responses to increase the frequency bandwidth [20,21,22,23]. Another popular approach is tuning the resonant frequency to match the ambient vibration by altering the effective spring stiffness of the cantilever beam. Wu et al. used a movable screw to adjust the gravity center of the proof mass and successfully tuned the resonant frequency for a range covering 130–180 Hz [24]. Liu et al. [25] used an additional cantilever stopper as a frequency-up-converter (FUC) that suppresses the vibration of a cantilever harvester with a lower resonance, increasing the operation bandwidth to 22 Hz at 0.8 *g*, where *g* denotes the standard acceleration due to the earth’s gravity, *g* = 9.80665 m/s^2^. A similar FUC mechanism that uses snap-through buckling has also been reported [26,27].

Another popular effort is the nonlinear energy harvester, where the system’s nonlinearity is exploited to improve the harvester’s performance [10,28]. Marzencki et al. developed a PEH device with a clamped–clamped boundary and a centrally located seismic mass and introduced nonlinearity by redesigning the interlayer stresses of the beam [29]. Many researchers have studied a bistable potential well that exploits nonlinearity induced by magnetic interactions in PEH, broadening the operational bandwidth and showing superior power generation [30,31,32]. These harvesters appear promising but still require the external involvement of magnets. Other authors proposed stress-induced monostable nonlinear PEHs using a doubly clamped beam to demonstrate that both bandwidth and performance of the nonlinear energy harvester are improved [29,33,34]. Wang et al. [35] investigated a tri-state nonlinear PEH by introducing a nonlinear magnetic force using four magnets. Podder et al. demonstrated that magnetic repulsion-induced bistability combined with stretching-induced quartic potential results in a broadband harvester but reduces the harvested output power [36]. Though the nonlinear harvester performs better than a linear harvester, the magnets make the device bulky, and the power density per device volume is relatively lower.

The problem with mesoscale devices is how to lower the operating frequency while producing sufficient displacement to generate considerable electric power. The authors attempted here to find an alternate solution to lowering the frequency, widening the operational bandwidth, and gaining more power output of a mesoscale piezoelectric harvester while studying the geometric nonlinearity of the PEH.

This paper presents a monostable nonlinear PEH which combines bending and stretching in an unconventional tapered fixed-guided spring. Finite Element Method (FEM) analysis was performed to study harvesting performance and nonlinearity, and was compared to conventional nonlinear PEHs with rectangular and tapered spring beams. The dynamic model predicts and compares the performance of all three harvesters. The proposed harvester has a compact design and is not affected by electromagnetic fields due to the absence of extra magnets and stoppers. This design of the harvester makes it convenient for low-frequency excitation harvesting and improves the performance by widening the bandwidth and increasing the harvested energy of the harvester.

The paper is arranged in the following order: Section 2 defines the design and modeling of the proposed harvester. FEM simulation demonstrates the initial structural analysis of the proposed harvester compared to conventional nonlinear PEHs. Section 3 explains the results from the analytical model analysis and compares the performance of different nonlinear PEH designs. The concluding comments are given in Section 4.

## 2. Design and Modeling

**Figure 1:**
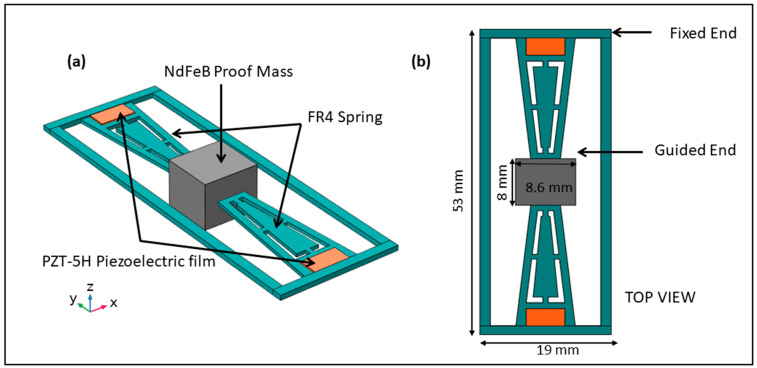
(**a**) Schematic diagram of the proposed nonlinear PEH with an exsect-tapered FR4 spring, and (**b**) top view of the nonlinear PEH.

**Figure 2:**
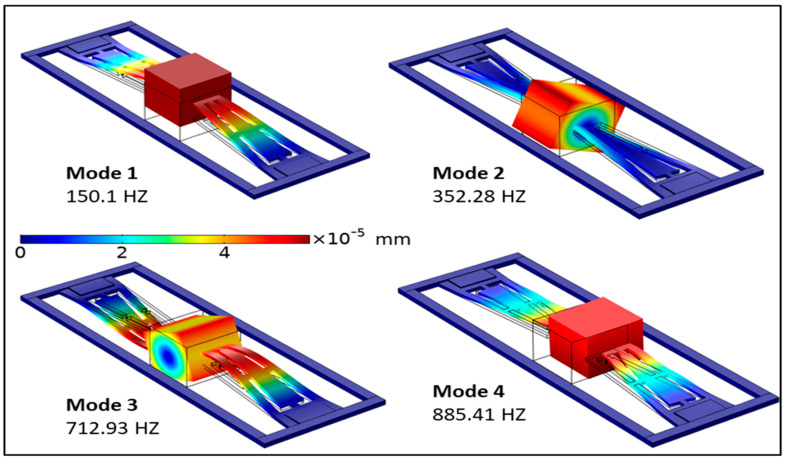
FEM analysis of the exsect spring structure design depicting the first four modes of the nonlinear PEH. The color legend shows the deformation of the structure in the *z*-axis.

Figure 1 depicts the schematic diagram of the proposed nonlinear PEH. The nonlinear harvester comprises an exsect-tapered spring geometry fixed on two opposite sides by a fixed outer frame. The outer frame holding the ends of the beam is fixed while the rest of the structure is free to move such that the proof mass guides the other ends of the beam at the center, which can now be considered a fixed-guided set of beams (spring structure). The beam spring is made of 0.5 mm-thick FR4 (woven fiberglass embedded in flame retardant epoxy resin), which aids in lowering the operating frequency owing to its low Young’s modulus. As shown in Figure 1a, a central NdFeB proof mass of 8 mm × 8 mm, for reducing the resonant frequency, is suspended at the guided end of both beams to offer an even mass distribution in the harvester. NdFeB material was selected for the proof mass to be consistent with prior literature, thus ensuring a proper comparative study [34,37,38]. The spring area near the fixed ends of the device is covered by two PZT-5H piezoceramic regions of 3 mm × 0.2 mm, which are electrically isolated from each other. The FEM simulation of the harvester shows that the four eigen-modes are at 150.1, 352.8, 712.93, and 351 Hz, where the first mode has out-of-plane motion, and the latter modes have tilt and flip modes (Figure 2).

To validate the performance improvement in the design concept, structural analysis was performed by Finite Element Method (FEM) simulation to investigate the performance of nonlinear PEHs with the rectangular (non-tapered) spring, simple linearly tapered spring, and the exsect-tapered spring. To compare the characteristics of the harvesters, the key geometry dimensions of the spring and piezoelectric film and materials of the structures were kept similar, as listed in Table 1. The outer FR4 frame is fixed while the rest of the structure is free to move. The piezoelectric films at the two fixed ends are all the same size.

**Figure 3:**
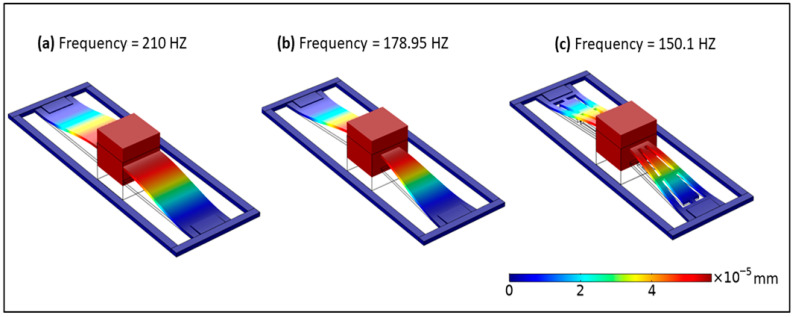
The resonant frequency of the nonlinear PEHs: (**a**) rectangular, (**b**) tapered, and (**c**) exsect-tapered.

**Figure 4:**
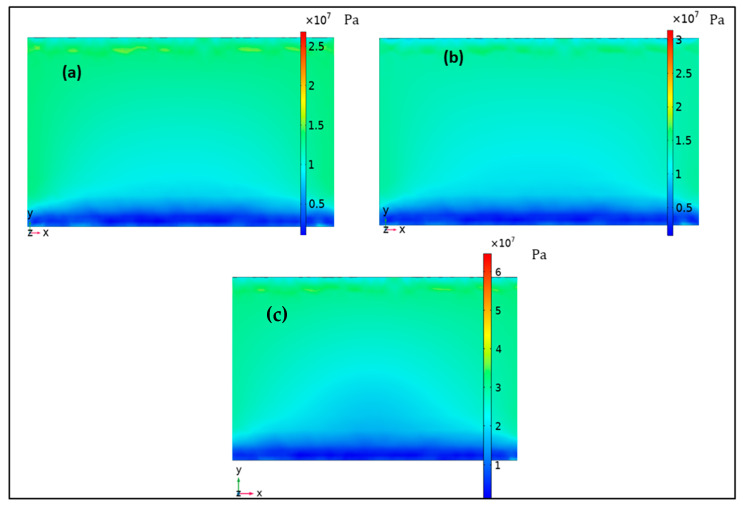
The distribution of stress in PZT-5H piezo film of the nonlinear PEHs: (**a**) rectangular, (**b**) tapered, and (**c**) exsect-tapered.

The first natural frequencies of the PEHs with rectangular, simple-tapered, and exsect-tapered springs are 210, 178.95, and 150.1 Hz, respectively, as shown in Figure 3. The exsect-tapered spring structure can reduce the operational frequencies of the PEH. Figure 4 demonstrates the stress distribution in the PZT-5H piezo film. The narrow width at the guided end of both the tapered structures enhances the stress when compared to the rectangular structure. It is observed that the exsect-tapered PEH experiences maximum stress. The increased stress in the exsect-tapered PEH will enhance energy generation due to the piezoelectric effect.

**Figure 5:**
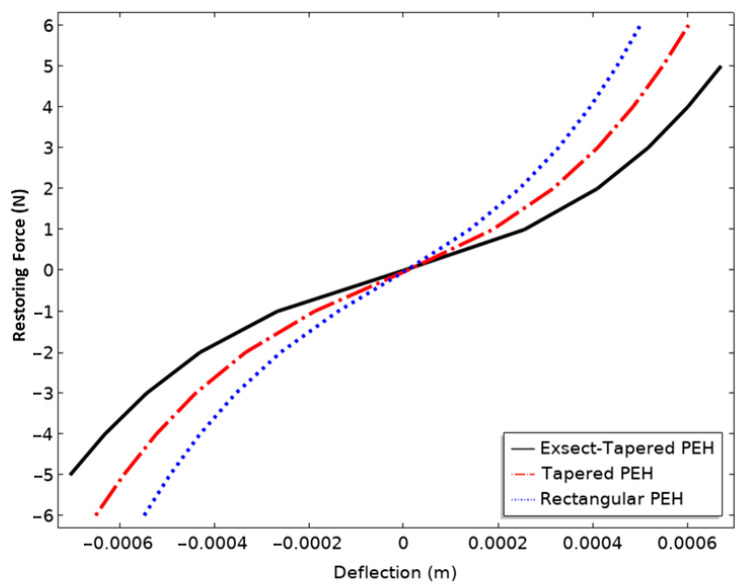
The restoring force of the spring vs. deflection amplitude of different nonlinear PEHs.

The average stress of each structure is summarized in Table 2. The nonlinear behavior of the proposed structure can be demonstrated by analyzing its mechanical stiffness based on the generalized restoring force [39],

(1) $$F\left(x\right)=k_{L}x+k_{NL}x^{3}$$

here F is the spring restoring force, $k_{L}$ is the linear stiffness constant due to bending of the FR4 spring, and $k_{NL}$ is the cubic stiffness constant due to stretching of the FR4 spring. Geometric nonlinearity is considered in the FEM model. The bending and stretching due to the large deformation of the structure causes nonlinear behavior. The stiffness coefficient constants $k_{L}$ and $k_{NL}$ were identified by finite element analysis using a Stationary Study by applying different body loads to the proof mass and measuring the corresponding relative deflections in the *z*-axis direction. Figure 5 shows the resulting force vs. displacement data, and the polynomial fitting technique in ORIGIN software (version 8.5.0, OriginLab Corporation, Northampton, MA, USA) was used to determine the stiffness coefficients $k_{L}$ and $k_{NL}$. For the initial deflection, the returning force of FR4 springs replicates the structure bending. After the deflection exceeds a certain value close to the thickness of the beam spring, the structure shows nonlinear behavior induced by stretching, and in that case, the spring restoring force involves both linear bending and stretching-induced components. The stiffness coefficients of all the PEHs are summarized in Table 1. It is observed that the coefficients $k_{L}$ and $k_{NL}$ of a conventional rectangular PEH are greater than those of the tapered PEHs, which accounts for its high frequency. The tapered PEHs have a difference in $k_{NL}$, but the $k_{L}$ of the exsect-tapered PEH is much smaller than that of the simple-tapered one. Therefore, the introduced exsect-tapered structure can reduce the operational frequency and provide sufficient nonlinear stiffness, which facilitates low and broadband ambient vibration harvesting. The nonlinearity degree can be increased by reducing the tapering ratio and thickness of the spring beams [40].

**Figure 6:**
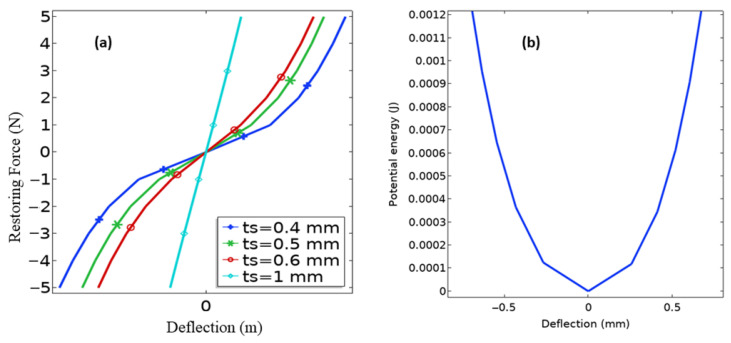
(**a**) The restoring force of spring vs. deflection amplitude corresponding to different spring thicknesses in the exsect-tapered PEH and (**b**) potential energy of the monostable PEH with a spring thickness ts = 0.5 mm.

Figure 6a shows the resulting nonlinear force–deflection relation for different spring thicknesses. In this case, an optimal thickness of 0.5 mm was selected for its sufficient nonlinearity, without compromising the stability of the structure owing to gravity load. Figure 6b shows that the potential energy function (U(x)=−Fdx) has one deep potential with its minimum at x=0, which leads to high energy intra-well oscillation that helps to achieve a wider operational bandwidth when compared to the conventional linear harvesters. Thus, the proposed system is a monostable nonlinear piezoelectric harvester based on the bending and stretching of the exsect-tapered spring beam design of the nonlinear PEH.

**Figure 7:**
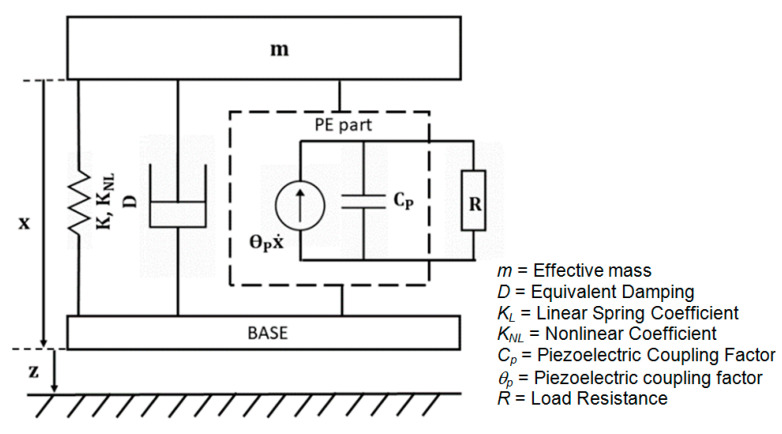
The physical lumped model of the nonlinear PEH.

The dynamic model of the nonlinear system can be approached as a lumped system of a mechanical vibration unit and a piezoelectric (PE) unit, as shown in Figure 7. Then, the differential equations governing the electromechanical coupling system are described by

(2) $$m\ddot{x}+D\dot{x}+K_{L}x+K_{NL}x^{3}+\theta_{p}V=m\ddot{z},$$

(3) $$C_{p}\dot{V}+\frac{V}{R}-\theta_{p}\dot{x}=0.$$

Here *m* is the effective mass, D=2 mξω is equivalent mechanical damping, ω is external frequency, $C_{p}$ is piezoelectric capacitance, $\theta_{p}$ is the piezoelectric coupling factor, V is piezoelectric voltage, and R is the optimal resistance of the considered PEH. The parameters in the equations (m, $K_{L}$ and $K_{NL}$) were identified through FEM analysis. The parameters and material properties of the nonlinear harvester models are detailed in Table 1. A MathWorks MATLAB (version 2020A, The MathWorks, Inc., Natick, MA, USA) single-step solver solved these ordinary differential equations s based on an explicit Runge-Kutta (4,5) formula and determined the generated electrical output voltage. For a voltage V, the output power harvested reaches a maximum, $P_{m}=\frac{V^{2}}{R}$ for an optimal load $R=\frac{1}{\omega C_{p}}$ [41]. The following section studies and compares the results of FEM simulations and the numerically simulated dynamic model of the harvesters.

## 3. Result and Discussion

**Figure 8:**
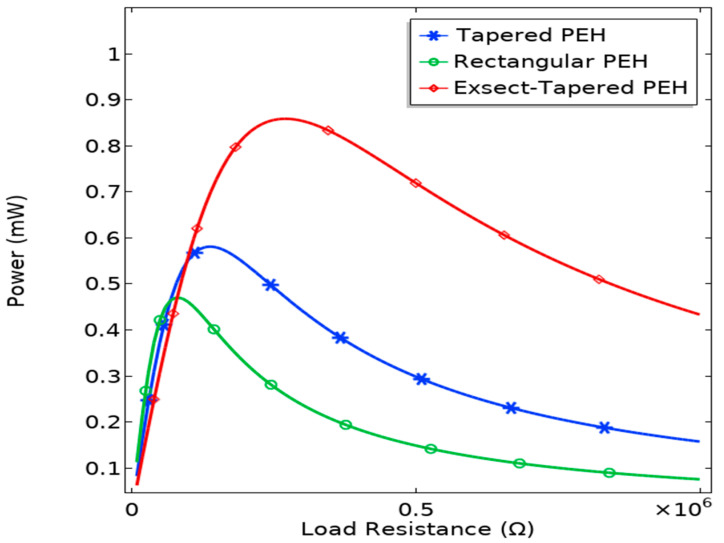
FEM-simulated output power of the nonlinear PEHs for varying load resistance.

The finite element model of the conventional rectangular, simple-tapered, and proposed exsect-tapered nonlinear PEHs were developed using COMSOL Multiphysics software (version 5.6, COMSOL Inc., Stockholm, Sweden) to find the optimal load resistance. The two PZT-5H films at the fixed ends are connected parallel to load resistance *R*. Under a constant 0.5 *g* input excitation, the parametric sweep of load resistances varied from 10 kΩ to 1 MΩ. Figure 8 shows the peak power output (RMS) for different load resistances for all three nonlinear PEHs. The maximum RMS powers generated for rectangular, tapered, and exsect-tapered nonlinear PEHs were obtained as 0.46, 0.58, and 0.88 mW, respectively, for corresponding optimal loads of 0.10, 0.14, and 0.27 MΩ, respectively. The output power generated by the exsect-tapered harvester is the highest among the three PEHs because of the maximum stress distribution in the piezo film.

**Figure 9:**
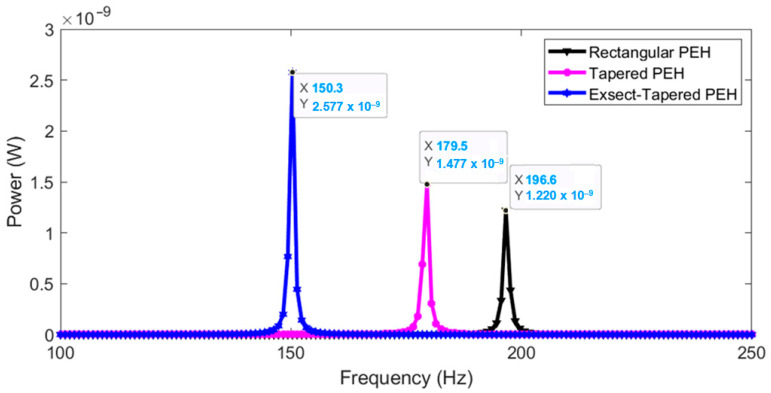
Numerically simulated frequency response (linear) of the PEHs for an input excitation of 0.001 *g*.

For low input excitation, the frequency response is almost a linear harvester since the stiffness coefficient $k_{L}$ is dominant when compared to the cubic coefficient $k_{NL}$; therefore, the nonlinear behavior of the response curves is not apparent. Figure 9 shows the response for a low input excitation of 0.001 *g*. The maximum RMS powers generated from the rectangular, tapered, and exsect-tapered PEHs are 1.22, 1.477, and 2.577 nW at 196.6, 178.5, and 150.3 Hz, respectively. The numerically simulated fundamental frequencies are close to the FEM simulated ones (210, 178.95, and 150.1 Hz). For a low input excitation of 0.001 *g*, the PEHs can be regarded as linear harvesters.

**Figure 10:**
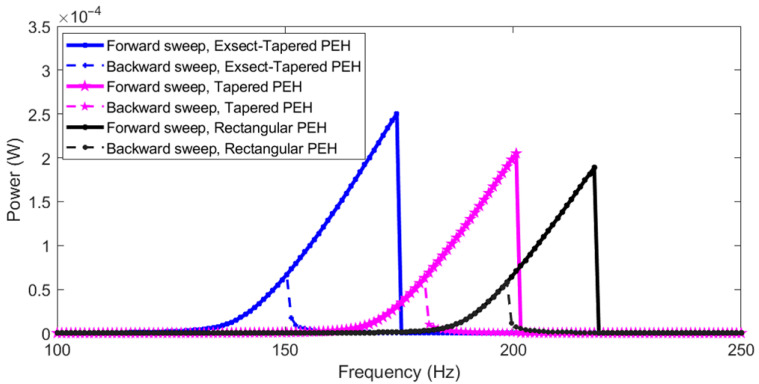
Numerically simulated frequency response (nonlinear) of the PEHs for an input excitation of 0.9 *g*. Solid lines represent forward sweep, and dashed lines represent backward sweep.

At higher levels of excitation, the cubic coefficient $k_{NL}$ becomes dominant due to stretching-induced nonlinearity. Figure 10 shows the frequency response for an input excitation of 0.9 *g*. As the excitation increases, the stress/strain experienced by the harvester intensifies, forcing it to stretch; the dynamic response of power output analyzed for frequency sweep in the forward (solid lines) and reversed direction (dashed lines) shows a hysteresis feature with the jumping phenomenon. The maximum RMS powers generated from the rectangular, tapered, and exsect-tapered PEHs were 1.8, 2.05, and 2.6 mW, respectively. The power output of the exsect-tapered was enhanced by 44.4 and 26.83% when compared to conventional rectangular and a simple linearly tapered, nonlinear PEH, respectively. The half-power bandwidths for rectangular, tapered, and exsect-tapered PEHs were 7.8, 8.1, and 9 Hz, respectively. The proposed exsect-tapered nonlinear PEH shows a bandwidth improvement of 15.38 and 11.11% when compared to a conventional rectangular and a simple linearly tapered, nonlinear PEH, respectively.

**Figure 11:**
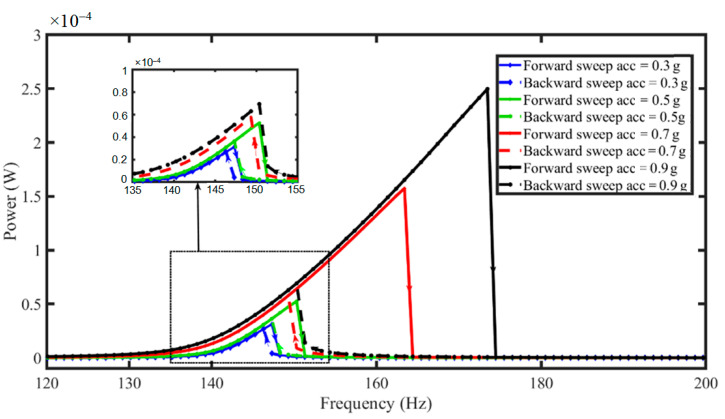
Analytical simulated output power for a varying frequency with load resistance R = 0.27 MΩ and different input excitations (acc) for forward (solid line) and backward (dash line) frequency sweeps in an exsect-tapered nonlinear PEH.

Figure 11 demonstrates the bandwidth widening due to increasing stretching-based nonlinearity when the applied excitation is increased in the exsect-tapered PEH. The maximum power outputs of the harvester were 0.032, 0.053, 0.16, and 0.24 mW for input excitations of 0.3, 0.5, 0.7, and 0.9 *g*, respectively. The central resonant frequency is calculated from the dynamic response as 153 Hz, as in reference [42]. An increased input excitation level improved both the bandwidth and output power harvested of the nonlinear PEH. The half-power bandwidth was found in the responses as being 2, 4, 7, and 9 Hz for input excitations of 0.3, 0.5, 0.7, and 0.9 *g*, respectively. Table 3 summarizes the resonant frequencies, optimal load, bandwidth, and output powers obtained by the different nonlinear PEHs.

The values of the comparison factor, NPD (Normalized Power Density) of the exsect-tapered nonlinear PEH, and the nonlinear PEHs reported earlier in the literature are listed in Table 4. The proposed harvester has a high NPD because of the high piezoelectric constant of PZT-5H used and lower operational frequency owing to the designed exsect-tapered fixed-guided spring structure with central proof mass. Also, our proposed energy harvester exhibits a considerably wide bandwidth. The exsect-tapered nonlinear PEH can enhance the output power generated and operational bandwidth for harvesting low ambient vibrations.

## 4. Conclusions

This paper proposes a monostable nonlinear Piezoelectric Energy Harvester (PEH) with an exsect-tapered spring beams. The harvester utilizes stretching-induced tension in fixed-guided spring beams to achieve a wider bandwidth and enhance the power output of ambient energy harvesting. FEM simulations were performed to demonstrate and validate the advantage of the harvester compared to conventional rectangular and simple tapered nonlinear PEHs with fixed-guided beams. The exsect-tapered spring beams with the same proof mass and dimensions as the other two nonlinear PEHs have the lowest linear stiffness coefficient; this enables its use for harvesting low-frequency vibrations. The proposed nonlinear PEH shows an improvement in bandwidth and power output when compared to the other two harvesters and reported nonlinear PEHs from literature. The harvester can be altered with different cutout designs of the exsect-tapered spring beams to lower the frequency further and tailor the nonlinearity degree to achieve a wider bandwidth and enhance the power output.

## Figures and Tables

**Table 1:** Material properties and parameters of the nonlinear PEH.

Description	Value
The effective mass of rectangular tapered and exsect-tapered PEH, m	4.1 *g*, 4.7 *g* and 3.781 *g*
Spring width at the fixed end	8.6 mm
Spring width at the guided end	4.3 mm
PZT-5H size	5.6 mm × 3 mm × 0.2 mm
$\mathrm{The}\;\mathrm{thickness}\;\mathrm{of}\;\mathrm{the}\;\mathrm{FR}4\;\mathrm{spring},\;t_{s}$	0.5 mm
$\mathrm{The}\;\mathrm{density}\;\mathrm{of}\;\mathrm{FR}4,\;\rho_{s}$	$1900\;(\mathrm{kg}/m^{3}$)
$\mathrm{Young}\;\mathrm{Modulus}\;\mathrm{of}\;\mathrm{FR}4,\;E_{s}$	22 (GPa)
$\mathrm{Young}\;\mathrm{Modulus}\;\mathrm{of}\;\mathrm{NdFeB},\;E_{n}$	160 (GPa)
$\mathrm{The}\;\mathrm{density}\;\mathrm{of}\;\mathrm{NdFeB},\;\rho_{n}$	$7800\;(\mathrm{kg}/m^{3}$)
$\mathrm{The}\;\mathrm{density}\;\mathrm{of}\;\mathrm{PZT}-5H,\;\rho_{p}$	$7500\;(\mathrm{kg}/m^{3}$)
$\mathrm{Young}\;\mathrm{Modulus}\;\mathrm{of}\;\mathrm{PZT}-5H,\;E_{p}$	64 (GPa)
$\mathrm{Piezoelectric}\;\mathrm{constant},\;d_{31}$	750 (pC/N)
$\mathrm{PZT}\;\mathrm{relative}\;\mathrm{Permittivity}\;\mathrm{constant},\;\varepsilon_{ss}$	39.84 (pF/m)
Damping ratio, *D*	0.003
Coupling coefficient	0.04156
$\mathrm{Piezoelectric}\;\mathrm{Capacitance},\;C_{p}$	5.65 (nF)
Load Resistance, *R*	0.27 MΩ

**Table 2:** Comparison of stiffness and stiffness coefficients of different PEHs.

PEH	Stress (MPa)	*k_L_* (N/m)	*k_NL_* (N/m^3^)
Rectangular	2.5	6847	1.73 × 10^10^
Tapered	3	4867	9.25 × 10^9^
Exsect-Tapered	6	3227	8.92 × 10^9^

**Table 3:** Comparison of output results for an input base excitation of 0.9 *g*.

Nonlinear PEH	Resonant Frequency (Hz)	Optimal Load (Ω)	Bandwidth (Hz)	$\mathrm{Piezoelectric}\;\mathrm{Power}\;P_{P}\;(\mathrm{mW}),\;\mathrm{acc}=0.9\;g$
Rectangular	196.6	1.0 × 10^5^	7.8	1.8
Tapered	179.5	1.4 × 10^5^	8.1	2.05
Exsect-Tapered	150.3	1.7 × 10^5^	9	2.6

**Table 4:** Comparison of reported nonlinear wideband piezoelectric energy harvesters.

S. No.	Wideband Harvester	Bandwidth (Hz)	Input Excitation (*g*)	Device Volume (cm^3^)	Generated Power Output (μW)	Normalized Power Density $\left(\mathrm{NPD}=\mu W/cm^{3}g^{2}\right)$
1.	Multimode [43]	59	0.5	0.0041	0.61	595.12
2.	FUC [25]	22	0.8	0.0161	0.19	18.43
3.	Clamped-Clamped [34]	9.64	0.1	1.22	125	10245
4.	Rectangular nonlinear (Fixed-Guided)	7.8	0.9	0.824	1800	2696.87
	Tapered nonlinear (Fixed-Guided)	8.1		0.779	2050	3248.86
	Exsect-Tapered (This Work)	9		0.753	2600	4262.78

## Data Availability

All data needed to evaluate the results presented in the paper are already included in the manuscript.
